# BMP4 Exerts Anti-Neurogenic Effect via Inducing Id3 during Aging

**DOI:** 10.3390/biomedicines10051147

**Published:** 2022-05-17

**Authors:** Tingting Li, Hongmei Liu, Dongfang Jiang, Keyan Yang, Jiaqi Shen, Haiping Feng, Sijia Wang, Yuxin Zhang, Yun Wang, Tie-Shan Tang

**Affiliations:** 1 State Key Laboratory of Membrane Biology, Institute of Zoology, University of Chinese Academy of Sciences, Chinese Academy of Sciences, Beijing 100101, China; litingting@ioz.ac.cn (T.L.); jiangdongfang@ioz.ac.cn (D.J.); yangkeyan@ioz.ac.cn (K.Y.); shenjiaqi@ioz.ac.cn (J.S.); fenghaiping@ioz.ac.cn (H.F.); wangsijia@ioz.ac.cn (S.W.); 15501120209@163.com (Y.Z.); wangyun@ioz.ac.cn (Y.W.); 2 Institute for Stem Cell and Regeneration, Chinese Academy of Sciences, Beijing 100101, China; 3 Beijing Institute for Stem Cell and Regenerative Medicine, Beijing 100101, China

**Keywords:** BMP4, Id3, adult neurogenesis, aging

## Abstract

Bone morphogenetic protein (BMP) signaling has been shown to be intimately associated with adult neurogenesis in the subventricular zone (SVZ) and subgranular zone (SGZ). Adult neurogenesis declines in aging rodents and primates. However, the role of BMP signaling in the age-related neurogenesis decline remains elusive and the effect of BMP4 on adult SVZ neurogenesis remains controversial. Here, the expression of BMP4 and its canonical effector phosphorylated-Smad1/5/8 (p-Smad1/5/8) in the murine SVZ and SGZ were found to be increased markedly with age. We identified Id3 as a major target of BMP4 in neuronal stem cells (NSCs) of both neurogenic regions, which exhibited a similar increase during aging. Intracerebroventricular infusion of BMP4 activated Smad1/5/8 phosphorylation and upregulated Id3 expression, which further restrained NeuroD1, leading to attenuated neurogenesis in both neurogenic regions and defective differentiation in the SGZ. Conversely, noggin, a potent inhibitor of BMP4, demonstrated opposing effects. In support of this, BMP4 treatment or lentiviral overexpression of Id3 resulted in decreased NeuroD1 protein levels in NSCs of both neurogenic regions and significantly inhibited neurogenesis. Thus, our findings revealed that the increased BMP4 signaling with age inhibited adult neurogenesis in both SVZ and SGZ, which may be attributed at least in part, to the changes in the Id3-NeuroD1 axis.

## 1. Introduction

Neurogenesis is a sequential and complex biological process which refers to the process where neural stem cells (NSCs) activate, proliferate, differentiate, mature, migrate and integrate into the existing neuronal circuitry to exert their functions [1]. It is widely acknowledged that neurogenesis continues and persists into adulthood in the central nervous system (CNS) of mammals, although adult neurogenesis in human remains controversial [2,3,4]. Adult neurogenesis in the subventricular zone (SVZ) of the lateral ventricle and the subgranular zone (SGZ) of the hippocampal dentate gyrus declines dramatically during aging [5,6], but the underlying mechanism leaves much to be explored [7]. Understanding the molecular and cellular regulation mechanisms of neurogenesis in aged brain will shed some light on interventions to achieve healthy aging.

Age-associated reduction in neurogenesis is attributed to either intrinsic decline in NSC function or the slow responsiveness of NSCs to environmental stimuli [8]. Compelling evidence supports the idea that adult neurogenesis is dynamically regulated by a number of signal pathways, such as Wnt/β-catenin pathway, sonic hedgehog (Shh) signaling, notch pathway, fibroblast growth factor (FGF) signaling, insulin-like growth factor-1 (IGF-1) signaling, BDNF/NGF-TrkB signaling and bone morphogenetic protein (BMP) signaling [9,10,11,12,13]. Interactions between different modules guarantee an elaborate and precise modulation of adult neurogenesis during aging [10,14,15,16]. In particular, an age-associated increase in BMP signaling in hippocampus contributes a lot to the decline of hippocampal neurogenesis [17]. However, how BMP signaling in the SVZ changes with age and its association with adult SVZ neurogenesis remains unclear.

Within the broad and heterogeneous BMP family, BMP4, is important for embryogenesis and development, and is also crucial in neural cell fate specification during adulthood and brain injury [18,19]. It is noteworthy that while the role of BMP4 in adult hippocampal neurogenesis has been consistent, showing inhibitory effects [20,21,22], the study of the effect of BMP4 signaling on adult SVZ neurogenesis leads to conflicting reports. BMP4 has been reported to inhibit cell proliferation in the SVZ in vitro [23,24] and in vivo [25], while ectopic expression of noggin, a BMP antagonist, promotes SVZ neurogenesis. However, there are also studies which indicate that BMP signaling positively regulates neurogenesis in the adult SVZ [26]. Although there is no definite conclusion of these variations, the potential roles of BMP4 signal for changes in neurogenesis have been speculated upon.

The inhibitor of differentiation/DNA binding (Id) proteins (Id1-4) are major downstream transcriptional targets of BMP signaling. They show distinct expression patterns in the adult brain and have been shown to promote stemness of NSCs [27]. For example, Id4 is the most widely expressed Id protein in hippocampus radial glia-like cells (RGLs) and dominates other Id members to induce NSC quiescence in a Smad4 independent manner [28], whereas Id3 is most strongly regulated by BMP-Smad4 signaling and is expressed mostly in progenitor cells in the SVZ and SGZ [28]. However, the contribution of Id proteins to the age-related decline of adult neurogenesis remains unclear.

Here, we revealed that BMP4 and its earliest responsive target p-Smad1/5/8 increased gradually with age. BMP4 activated Smad1/5/8 phosphorylation and upregulated Id3 expression, leading to attenuated neurogenesis of both neurogenic regions and impaired newborn neuron development in the SGZ. Id3 acted as a repressor of the downstream transcription factor NeuroD1, to inhibit neuronal differentiation with age. These findings provide important insights into the central role of canonical BMP4 signaling via Id3-NeuroD1 in the regulation of adult SVZ and SGZ neurogenesis during aging.

## 2. Materials and Methods

### 2.1. Cell Lines

293T (ATCC, Cat#CRL-3216, Manassas, VA, USA) and C17.2 (Meisen CTCC, Cat#CTCC-400-0350, Panan, China) cell lines were cultured in DMEM medium (GIBCO, Cat#12100046, Paisley, UK) supplemented with 10% FBS (GIBCO, Cat#10439024, Paisley, UK), 100 U/mL penicillin, and 100 μg/mL streptomycin (GIBCO, Cat#10378016, Paisley, UK) at 37 °C in 5% CO_2_. All cell cultures tested negative for mycoplasma contamination.

### 2.2. Animal Ethics Statement

Mouse maintenance and handling complied with the Chinese Institute of Zoology’s Guidelines for the Care and Use of Laboratory Animals, and strictly obeyed the rules of the Regulations for the Care and Use of Laboratory Animals by the Ministry of Science and Technology of China. All animal experiments met the standards of the Animal Care and Use Committee at the Institute of Zoology, Chinese Academy of Sciences (Permission Number: IOZ18009, 8 March 2018).

### 2.3. Animals

Wild-type (WT) female C57BL/6N mice at different stages of senescence (2–28 months old (MO)) were chosen. In particular, 2, 8, 12 and 18 MO female mice were used in immunofluorescence staining and in situ hybridization experiments, while 2, 8, 14 and 28 MO mice were used for Western blotting. All animals were kept under SPF conditions under the same feeding conditions, temperature and humidity with free access to food and water. The mice were maintained under standard conditions of 12/12 h of light/dark at 25 °C before and after surgery.

### 2.4. Drugs, Antibodies and Devices

The following drugs were used. Recombinant human noggin (PeproTech, Cat#120-10C, Matmata, Rehovot, Israel), recombinant human BMP4 (PeproTech, Cat#120-05, Matmata, Rehovot, Israel), BrdU (Roche, Cat#10280879001, Metroplaza, Hongkong, China).

The following primary antibodies were used. Chicken anti-nestin (Abcam, Cat#ab134017, RRID: AB_2753197, Waltham, MA, USA), rat anti-BrdU (Abcam, Cat#ab6326, RRID: AB_305426, Waltham, USA), rabbit anti-BMP4 (Abcam, Cat#ab39973, RRID: AB_2063523, Waltham, MA, USA), rabbit anti-SMAD1/5/8 (Abcam, Cat#ab66737, RRID: AB_2192755, Waltham, MA, USA), mouse anti-NeuroD1 (Abcam, Cat#ab60704, RRID: AB_943491, Waltham, MA, USA), rabbit anti-doublecortin (Cell Signaling Technology, Cat#4604, RRID: AB_561007, Danvers, CA, USA), rabbit anti-phospho-Smad1 (Ser463/465)/Smad5 (Ser463/465)/Smad8 (Ser465/467) (Cell Signaling Technology, Cat#13820, RRID: AB_2493181, Danvers, CA, USA), rabbit anti-BMP4 (Santa Cruz, Cat#SC-12721, RRID: AB_2063534, Dallas, TX, USA), mouse anti-Id3 (Santa Cruz, Cat#SC-56712, RRID: AB_783921, Dallas, TX, USA), mouse anti-BMP4 (Santa Cruz, Cat#SC-12721, RRID: AB_2275404, Dallas, TX, USA), mouse anti-GAPDH (Proteintech, Cat#60004-1-Ig, RRID: AB_2107436, Wuhan, Hubei), mouse anti-actin (Proteintech, Cat#66009-1-lg, RRID: AB_2687938, Wuhan, Hubei), rabbit anti-ID3 (Proteintech, Cat#10389-1-AP, RRID: AB_2248830, Wuhan, Hubei), mouse anti-GFAP (Chemicon, Cat#MAB360, RRID: AB_11212597, Pasir Panjang, Singapore), rabbit anti-phospho-Smad1/Smad5/Smad8 (Ser463/465) (Millipore, Cat#AB3848, RRID: AB_177439, Hayward, CA, USA), sheep anti-Digoxigenin-AP, Fab fragments (Roche, Cat# 11093274910, RRID: AB_514497, Metroplaza, Hongkong, China).

The sources of devices used were as follows: Alzet Brain Infusion Kit 3 (Alzet, Cat#0008851, Cupertino, San Francisco, CA, USA), Alzet Model 1007D (Alzet, Cat#0000290, Cupertino, San Francisco, CA, USA), RNeasy Mini Kit for RNA purification (Qiagen, Cat#151040629, Boston, MA, USA).

### 2.5. Brain Tissue Processing

Mice were anesthetized, perfused transcardially with PBS and 4% paraformaldehyde (PFA). Brains were removed and fixed further in 4% PFA overnight and then immersed in 30% sucrose for 24–48 h. The brains were then imbedded in O.C.T. Compound (Tissue-Tek, Cat#4583, Torrance, Los Angeles, CA, USA) and coronal sections at 20 μm were cut via a cryostat (Leica, Cat#CM1950, Wetzlar, Germany). Sections were stored in a glycerol-based cryoprotectant (glycerol, ethylene glycol, and 0.1 M phosphate buffer, pH 7.4, 1:1:2 by volume) at −20 °C until further immunostaining analysis.

### 2.6. Protein Isolation and Western Blotting

For protein dynamics analysis, 2, 8, 14 and 28 MO female mice were used. Three mice were used in each group. The SVZ and SGZ were dissociated from fresh mouse brain and lysed with RIPA lysis buffer (50 mM Tris-HCl, pH 8.0, 150 mM NaCl, 0.1% SDS, 1% NP-40, 0.5% sodium deoxycholate added with 1 mM EDTA, 1 mM NaF, 1 mM EDTA, 1 mM PMSF, 50 mM DTT and 1× protein inhibitor cocktail). Protein samples were boiled for 10 min in strong denaturing conditions and loaded onto polyacrylamide gels for SDS-PAGE. Gels were then transferred onto polyvinylidene difluoride membranes at 4 °C for the required time, after which the membranes were immersed in TBST with 10% nonfat dry milk for 1 h at room temperature. The membranes were incubated with primary antibodies diluted in 5% nonfat dry milk in TBST overnight at 4 °C. After washing in TBST for 30 min, the membranes were incubated with HRP-linked anti-rabbit IgG and HRP-linked anti-mouse IgG secondary antibodies for 2 h. After washing in TBST for 40 min, the membranes were developed using Super Signal West Pico enhanced chemiluminescent reagent (Thermo Scientific, Cat#34580, Hongkong, China).

### 2.7. Quantitative RT-PCR

Cells were lysed with Trizol reagent. RNA was extracted using Direct-zol RNA MiniPrep Kit (Zymo Research, Cat#R2052, Costa Mesa, CA, USA) according to manufacturer’s instructions. Total RNA was reverse transcribed into complementary DNA using GoScript Reverse Transcription System (Promega, Cat#A5003, Fitchburg, Wisconsin, WI, USA). Quantitative real-time PCR was performed using SYBR Green QPCR Master Mix (Invitrogen, Cat#K0251, Carlsbad, CA, USA) in 20 μL reaction volume. Thermal cycling was performed on the CFX Connect Real-Time PCR Detection System (Bio-Rad, Cat#1855201, Hercules, CA, USA). Three independent experiments were performed, and gene expression was calculated relative to endogenous control GAPDH. Primer sequences were as follows (Table 1).

### 2.8. In Situ Hybridization

A 399 bp mouse cDNA of BMP4 was used as a template for an in situ hybridization probe. The digoxigenin-labeled BMP4 antisense oligonucleotide probe was synthesized by an in vitro transcription kit from Promega. Primers used were as follows: Fd: 5′-GGAGCGGAGGCTGTAAGTTT-3′; Rs: 5′-ACCTCCCTGTCGCAAGAATG-3′. Fresh 10 μm slide-mounted coronal cryostat sections of 2, 8, 12 and 18 MO female mice were collected and fixed in 4% PFA for 5 min, then washed with PBS. The section was digested in proteinase K (10 μg/mL dissolved in 50 mM Tris-HCl, pH 8.0, 5 mM EDTA) for 10–15 min at room temperature. After a second round of PFA fixation and PBS washing, the sections were then incubated in pre-hybridization buffer (50% formamide, 5× salt sodium citrate buffer, pH 7.0, 0.25 mg/mL salmon sperm DNA, 0.5 mg/mL yeast tRNA, 5× Denhardt’s solution) for 4–6 h at 56 °C. Then, the digoxigenin (DIG)-labeled BMP4 antisense oligonucleotide probe was added in pre-hybridization buffer and incubated at 56 °C overnight. The next day, sections were washed in SSC and Tris-HCl/saline buffer (100 mM Tris–HCl, pH 7.5, 150 mM NaCl), incubated with 10% heat-inactivated sheep serum (HISS) in Tris-HCl/saline buffer for 1 h, followed by anti-digoxigenin antibody (Roche, Germany, 1:2000) incubation at 4 °C overnight. On day 3, after equilibrating the sections with freshly prepared NTMT buffer (100 mM Tris-HCl, pH 9.5, 100 mM NaCl, 50 mM MgCl_2_) for 5–10 min, the hybridization process was developed with BM Purple AP Substrate (Roche, Germany) at room temperature for 2–24 h in the dark chamber. The signals were checked periodically and reaction stopped with PBS/EDTA buffer. Sections were observed using an optical microscope (Leica Aperio VESA, Wetzlar, Germany).

### 2.9. Immunofluorescence

For immunofluorescence of cells, after cultured C17.2 cells were treated with 100 ng/mL BMP4 for 12 h, the cells were fixed with 4% PFA for 10 min at room temperature and washed with PBS. Then immersed in 0.2% TritonX-100 for 10 min and blocked with 10% donkey serum for 1 h, before being incubated with mouse anti-Id3 antibody (1:300) overnight at 4 °C, followed by Alexa Fluor donkey anti-mouse 488 nm antibody (1:1000) for 1 h.

For fluorescence staining of tissues, free-floating frozen sections were removed from the cryoprotectant buffer and washed thoroughly in PBS for 60 min. Sections were then kept in blocking buffer (PBS with 5% (*v*/*v*) normal donkey serum, 1% (*w*/*v*) BSA and 0.5% (*v*/*v*) Triton X-100) for 1 h at room temperature on a shaker. Then, sections were incubated with the primary antibodies in diluted buffer (PBS with 2.5% (*v*/*v*) normal donkey serum, 0.5% (*w*/*v*) BSA and 0.25% (*v*/*v*) Triton X-100) at room temperature for 30 min followed by 4 °C overnight. The next day, sections were washed in TBST (pH 7.6) for 3 × 15 min and incubated with secondary antibodies in diluted buffer for 2 h at room temperature. Then, sections were washed 3 × 15 min in TBST (pH 7.6), followed by the fluorescent nuclear dye Hoechst 33342 for 5 min, then mounted with mounting medium and kept at 4 °C until further microscopic analysis. Confocal single plane images and Z stacks were taken with a Nikon confocal microscope (Minato, Tokyo, Japan) equipped with four lasers of wavelengths 405, 488, 561 and 647 nm.

### 2.10. Recombinant Lentivirus Production

Lentivirus are produced in HEK293T cells using a second generation lentiviral system. Envelope plasmid pMD2.G, packaging plasmids psPAX2 and shRNA plasmid expressing mCherry-tagged protein in the ratio of 1:3:4 ratio were co-transfected into 293T cells using PEI. The supernatants from three 10 cm dishes were collected 60 h post-transfection and filtered through a 0.45 μm filter. Viruses were concentrated by ultracentrifugation (100,000× *g* for 2 h) at 4 °C. The virus pellets were then washed twice with cold PBS and resuspended in 15 μL PBS. We usually obtained 1 × 10^9^ infectious lentiviral particles/mL.

### 2.11. In Vivo Overexpression via Lentivirus Stereotaxic Injections to SVZ or SGZ

Mice at the age of 9 MO were anesthetized and viruses were stereotaxically injected into the SVZ or SGZ with equal volume and similar titer of lentivirus, with 1.5 μL of LV-NC on the left hemisphere and 1.5 μL of LV-WT-Id3 on the right hemisphere. Mice were given two injections within ten days. The coordinates of intracerebroventricular injection to the left lateral ventricle were as follows: antero-posterior = 0.5 mm relative to bregma; lateral = −1.6 mm to the midline; and depth = 2.9 mm down from the surface of the skull. The coordinates of the dentate gyrus were as follows: anteroposterior: −2.3 mm relative to bregma; lateral: ±1.6 mm to the midline; depth: 2.3 mm down from the surface of the skull. Mice were allowed to recover for 7 days, followed by BrdU intraperitoneal injections (50 mg/kg body weight) and analyzed 24 h post-injection.

### 2.12. Osmotic Pump Grafting

Infusion of mice was performed using an Alzet mini-osmotic pump. The cannula (Alzet, Brain Infusion Kit III) was implanted stereotaxically in the left lateral ventricle (antero-posterior, 0.5 mm; lateral, −1.3 mm; depth, 2.9 mm relative to bregma and the surface of the brain). Three different concentrations of BMP4 and noggin were continuously infused into the brain for 7 days, with vehicle solution (0.9% NaCl) as a control. Low, medium and high doses of BMP4 were prepared at final concentrations of 300, 500 and 800 ng/d, while noggin was at 150, 300 and 500 ng/d, respectively.

### 2.13. BrdU Labeling Experiments

For analysis of cell proliferation, C17.2 cells were pretreated with BMP4 (100 ng/mL) for 12 h, then incubated in the BrdU labeling solution (10 μmol) for 2 h at 37 °C. Cells were then fixed in PFA and washed with PBS. After acidification in 1N HCl at room temperature for 45 min and washing with PBS several times, cells were immersed in 0.2% TritonX-100 for 10 min and blocked with 10% donkey serum for 1 h. Cells were incubated with rat anti-BrdU antibody (1:1000) overnight at 4 °C, followed by incubation with Alexa Fluor donkey anti-rat 488 nm antibody (1:1000) for 2 h.

For analysis of cell proliferation in the adult mouse brain, adult mice were given 3 injections of BrdU (50 mg/kg body weight, i.p.) within 24 h, and the animals were sacrificed 24 h later followed by transcardial perfusion with cold PBS and 4% PFA. Brains were removed and processed for OCT embedding. For quantification of BrdU-positive cells in the adult brain, 20 μm free-floating sections were treated with 2N HCl at 37 °C for 30 min and neutralized by incubation with 0.1 M sodium borate buffer (pH 8.5) for 10 min at room temperature. Sections were blocked in 5% donkey serum in Tris-buffered saline plus 0.5% Triton X-100 for 1 h, and subsequently incubated with rat anti-BrdU antibody (1:500) overnight at 4 °C, followed by Alexa Fluor donkey anti-rat 488 nm antibody (1:1000) for 2 h. Proliferating SVZ cells located in the cell dense region around the ventricle were quantified. At least three sections from each animal were evaluated.

### 2.14. Microscopy and Imaging

Confocal single plane images were taken with a Nikon N-SIM S (Minato, Tokyo, Japan) with corresponding laser lines (405, 488, 561 and 647 nm) using 40×, 20× and 10× objective lens. For dendritic branching analyses, 30 μm floating brain sections and a 40× objective lens were used. Z stacks of DCX-positive neurons dendrites were captured at 3 μm intervals and analyzed by ImageJ software (NIH, Bethesda, MD, USA). Data were further extracted for total dendritic length and Sholl analysis. A total of 20–25 neurons per group from 3 different animals were analyzed. The exact value of *n* is described in the figure legends.

### 2.15. Statistical Analysis

All data were presented as mean ± SEM unless stated otherwise. Statistical significances of data were analyzed using Student’s paired, and unpaired *t* tests. Data from multiple groups were analyzed with two-way analysis of variance (ANOVA) followed by Tukey’s multiple comparisons test. *N* represents the number of evaluated animals. Significance was set at *p* < 0.05 (*), *p* < 0.01 (**), *p* < 0.001 (***).

## 3. Results

### 3.1. Canonical BMP Signaling Increases with Age in Both Neurogenic Regions

**Figure 1:**
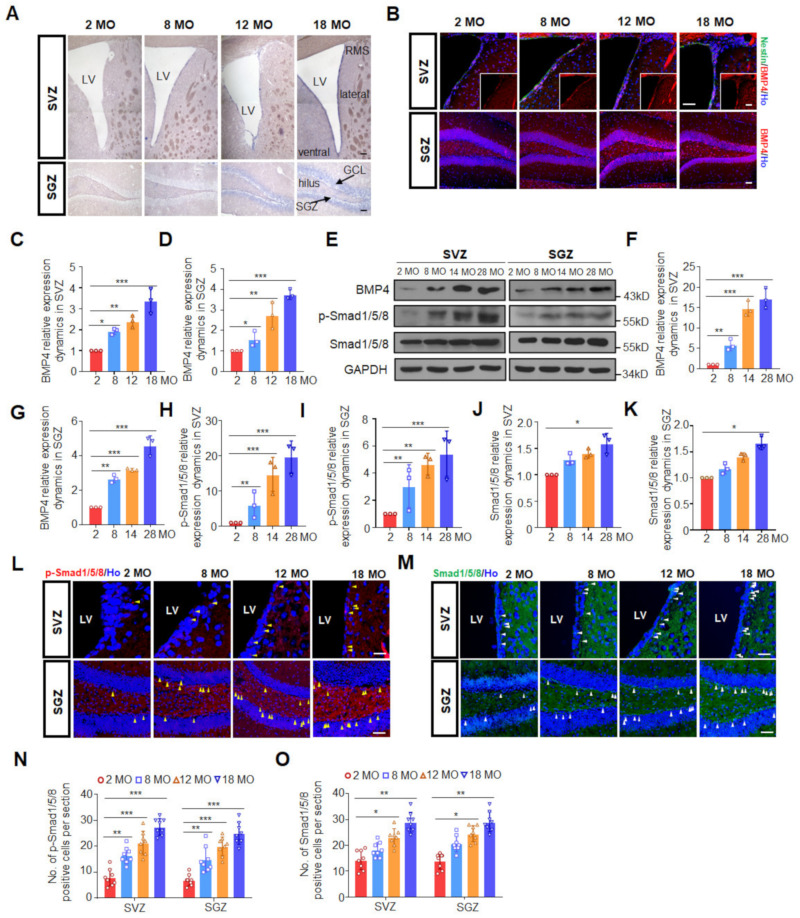
Canonical BMP4 signaling increases during aging. (**A**) In situ hybridization analysis of BMP4 mRNA in the SVZ (top panel, scale bar, 100 μm) and SGZ (bottom panel, scale bar, 50 μm) of female mice at 2, 8, 12 and 18 MO. (**B**) Representative images of BMP4 expression in the SVZ and SGZ of female mice at 2, 8, 12 and 18 MO. Scale bar, 50 μm. (**C**) BMP4 relative expression dynamics in the SVZ of female mice at 2, 8, 12 and 18 MO. * *p* < 0.05, ** *p* < 0.01 and *** *p* < 0.001. (**D**) BMP4 relative expression dynamics in the SGZ of female mice at 2, 8, 12 and 18 MO. * *p* < 0.05, ** *p* < 0.01 and *** *p* < 0.001. (**E**) Western blotting analyses of proteins extracted from the SVZ and the SGZ of three mice at 2, 8, 14 and 28 MO. GAPDH is used as a loading control. (**F**–**K**) Relative quantification of Western blotting analysis of protein levels in the SVZ and SGZ of female mice at 2, 8, 14 and 28 MO: (**F**) BMP4 in the SVZ; (**G**) BMP4 in the SGZ; (**H**) p-Smad1/5/8 in the SVZ; (**I**) p-Smad1/5/8 in the SGZ; (**J**) Smad1/5/8 in the SVZ; (**K**) Smad1/5/8 in the SGZ. Data is represented as the mean protein intensity normalized to GAPDH ± S.E.M. from 3 independent mice. * *p* < 0.05, ** *p* < 0.01 and *** *p* < 0.001. (**L**) Representative images of p-Smad1/5/8 expression in the SVZ (top panel, scale bar, 25 μm) and SGZ (bottom panel, scale bar, 50 μm) of female mice at 2, 8, 12 and 18 MO. Arrowheads refer to p-Smad1/5/8 positive signals. (**M**) Representative images of Smad1/5/8 expression in the SVZ (top panel, scale bar, 25 μm) and SGZ (bottom panel, scale bar, 50 μm) of female mice at 2, 8, 12 and 18 MO. Arrowheads refer to Smad1/5/8 positive signals. (**N**) Quantification of p-Smad1/5/8 positive cells in the SVZ and SGZ of female mice at 2, 8, 12 and 18 MO. ** *p* < 0.01 and *** *p* < 0.001. (**O**) Quantification of Smad1/5/8 positive cells in the SVZ and SGZ of female mice at 2, 8, 12 and 18 MO. * *p* < 0.05 and ** *p* < 0.01.

To monitor BMP4 expression dynamics during aging, in situ hybridization, Western blotting and immunofluorescence staining were performed to detect the level of BMP4 in the SVZ and SGZ from female mice of different ages. As shown in Figure 1A, BMP4 mRNA was expressed mainly in the dorsal, lateral and ventral walls of the lateral ventricle where NSC and progenitor cells were located, while in the hippocampus, intense identification of BMP4 mRNA was detected in hilus and dentate gyrus where NSC and granule neurons were located. Both mRNA transcripts (Figure 1A) and protein expression levels (Figure 1B–G) of BMP4 in the SVZ and SGZ displayed a progressive increase during aging. Although Western blotting showed that the increase in BMP4 in the aged SGZ was not as dramatic as that in the SVZ, the protein level indeed increased with age in both regions. Compared to 2 MO, the level of BMP4 in the 28 MO female mice showed a five-fold increase in the SGZ while the increase in the SVZ was much higher than that in the SGZ (Figure 1E–G).

We further detected the canonical BMP4 downstream signals by immunohistochemistry and found that the expression of Smad1/5/8 and its active form p-Smad1/5/8 showed a steady increase in both SVZ and SGZ (Figure 1L–O). Consistently, Western blotting results showed that the expression level of p-Smad1/5/8 increased markedly with age in both SVZ and SGZ (Figure 1E,H,I) whilst the baseline Smad1/5/8 expression showed only a slight increase (Figure 1E,J,K). It was noteworthy that although the immunohistochemistry and Western blotting revealed different fold changes of increases with age, which we speculated to be caused by the differences in the target events detection between these two techniques, the increase in p-Smad1/5/8 with age was greater than that of Smad1/5/8 in both neurogenic regions (Figure 1E,H–K), suggesting that Smad1/5/8 dynamics was just a part of the reason causing the increased p-Smad1/5/8 during aging. Other factors, such as members of the BMP family (BMP2, BMP4, BMP6, BMP7 and BMP9), might be involved, and the exact underlying mechanism remains to be explored [17,29]. Taken together, these results implicated the canonical BMP signal which exhibited a definite increased pattern during the aging process, encouraging us to explore what role the BMP signal pathway played and how it functioned with aging.

### 3.2. Increased BMP Signaling Impairs Adult Neurogenesis in SVZ and SGZ

**Figure 2:**
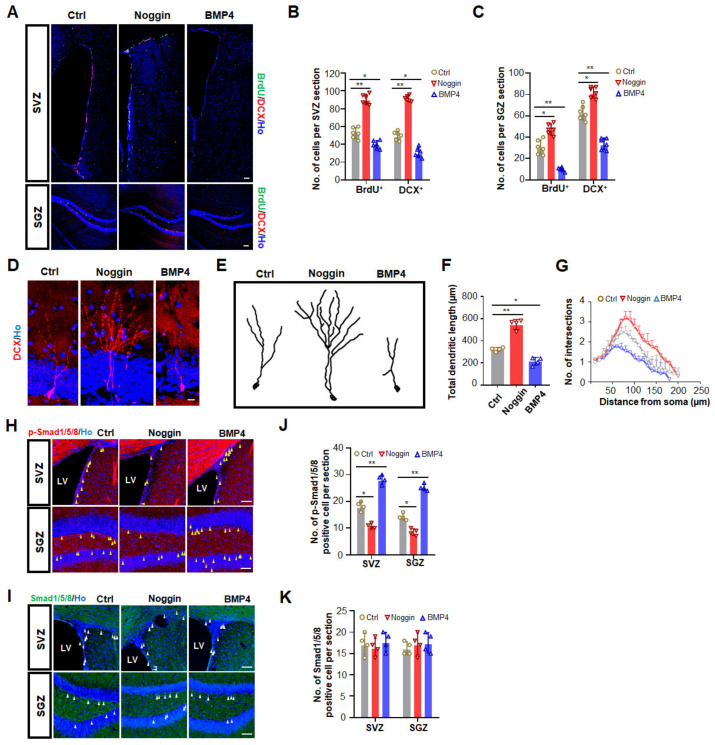
Canonical BMP4 signal inhibits adult neurogenesis in the SVZ and SGZ. (**A**) Representative images of BrdU and DCX positive cells in the SVZ and SGZ of 8 MO female mice one week after saline, a medium dose of noggin or BMP4 infusion. Scale bar, 100 μm. (**B**) Quantification of BrdU and DCX positive cells in the SVZ from 8 MO female mice one week after saline, medium dose of noggin or BMP4 infusion. * *p* < 0.05 and ** *p* < 0.01. (**C**) Quantification of BrdU and DCX positive cells in the SGZ from 8 MO female mice one week after saline, a medium dose of noggin or BMP4 infusion. * *p* < 0.05 and ** *p* < 0.01. (**D**) Representative images of the morphology of DCX positive immature neurons in the SGZ of 8 MO female mice one week after saline and a high dose of noggin or BMP4 infusion. Scale bar, 10 μm. (**E**) Image tracing of DCX positive immature neurons in the SGZ of 8 MO female mice one week after saline and a high dose of noggin or BMP4 infusion. (**F**) Quantification of the total dendritic length of DCX positive immature neurons in the SGZ of 8 MO mice one week after saline, a high dose of noggin or BMP4 infusion. *n* = 20–25 neurons from 3 mice. * *p* < 0.05 and ** *p* < 0.01. (**G**) Quantification of dendritic complexity of DCX positive immature neurons in the SGZ of 8 MO mice one week after saline, a high dose of noggin or BMP4 infusion. *n* = 20–25 neurons from 3 mice. (**H**) Representative images of p-Smad1/5/8 staining in the SVZ and SGZ of 8 MO female mice one week after saline, a high dose of noggin or BMP4 infusion. Scale bar, 50 μm. Arrowheads refer to p-Smad1/5/8 positive signals. (**I**) Representative images of Smad1/5/8 staining in the SVZ and SGZ of 8 MO female mice one week after saline, a high dose of noggin or BMP4 infusion. Scale bar, 50 μm. Arrowheads refer to Smad1/5/8 positive signals. (**J**) Quantification of p-Smad1/5/8 positive cells in the SVZ and SGZ from 8 MO female mice one week after saline, a high dose of noggin or BMP4 infusion. * *p* < 0.05 and ** *p* < 0.01. (**K**) Quantification of Smad1/5/8 positive cells in the SVZ and SGZ from 8 MO female mice one week after saline, a high dose of noggin or BMP4 infusion.

It is widely accepted that BMP4 inhibits adult hippocampal neurogenesis both in vitro and in vivo [19,21,30]. However, there are conflicting reports on the effect of BMP4 on adult neurogenesis in the SVZ, and its affected downstream effector in this process remains unknown. Given that different levels of BMP exert different effects, to clarify the effect of BMP4 on adult neurogenesis in vivo, three different doses of BMP4 were administered by intracerebroventricular (i.c.v) infusion into the left lateral ventricle of 8–9 MO mice via osmotic minipumps. Noggin, a potent BMP inhibitor, was also infused. Compared to the vehicle group, BMP4 immunostaining increased dramatically after BMP4 infusion but decreased greatly after noggin infusion (Supplemental Figure S1A), confirming the delivery efficiency of these proteins. The in vivo neurogenesis was then assessed by the incorporation of BrdU and staining of DCX in the SVZ and SGZ. Compared to the vehicle-infused mice, all three different doses of BMP4 exerted inhibitory effects on adult neurogenesis in both the SVZ and SGZ (Figure 2A). A medium dose of BMP4 led to decreases in the number of BrdU^+^ cells (SVZ: ~47%; SGZ: ~64%) and DCX^+^ cells (SVZ: ~44%; SGZ: ~37%) in both neurogenic regions (Figure 2B,C). Conversely, noggin counteracted these decreases, exhibiting increases in the number of BrdU^+^ cells (SVZ: ~50%; SGZ: ~43%) and DCX^+^ immature neurons (SVZ: ~58%; SGZ: ~30%) in the SVZ and SGZ (Figure 2B,C). Taken together, our data demonstrated that BMP4 inhibited adult neurogenesis in both SVZ and SGZ in a dose-dependent manner, with higher concentrations exerting stronger inhibitory effects.

We next investigated whether BMP4 infusion affects neurogenic differentiation in the SGZ. Shorter dendrites and less dendritic complexity of DCX^+^ newborn neurons were observed when high (Figure 2D–G) but not low (Supplemental Figure S1D,F) or medium (Supplemental Figure S1E,G) doses of BMP4 were infused into mice. By contrast, the dendritic arborization of newborn neurons in noggin-treated mice at three doses was significantly higher compared with those in vehicle-treated mice (Figure 2D and Supplemental Figure S1B). These results suggested that besides neurogenesis, BMP4 signaling also led to impaired dendritic arborization of newly generated neurons in the SGZ.

Levels of p-Smad1/5/8 were increased after BMP4 infusion but decreased after noggin infusion (Figure 2H,J), whereas the baseline Smad1/5/8 level remained consistent after both treatments (Figure 2I,K). These results suggested that BMP4 successfully induced canonical BMP signal activation, and the expression of Smad1/5/8 was not affected by the BMP4 level. It is not clear what causes the decline of Smad1/5/8 levels with age, which deserves further exploration.

### 3.3. BMP4 Inhibited Adult Neurogenesis via Up-Regulating Id3

**Figure 3:**
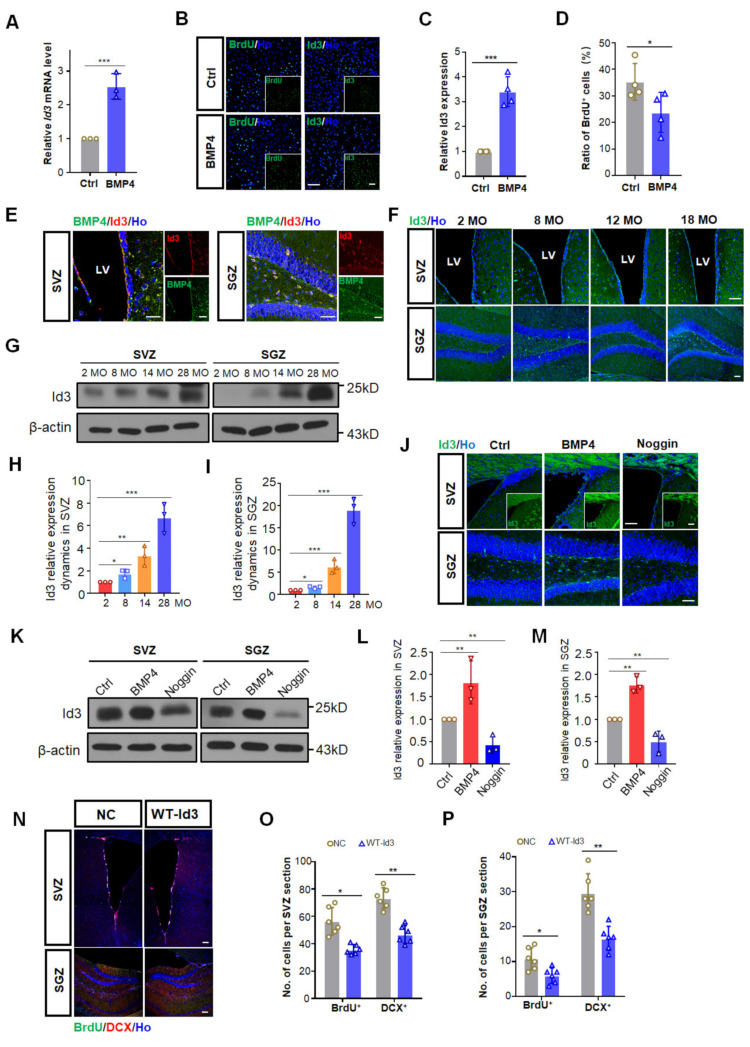
Id3 acted as an effector of canonical BMP signal during aging. (**A**) Transcription level of Id3 in ctrl and BMP4 treated C17.2 mouse stem cells analyzed by qPCR. *n* = 3. *** *p* < 0.001. (**B**) Representative images of Id3 and BrdU immunostaining in ctrl and BMP4 treated C17.2 mouse stem cells. Scale bar, 100 μm. (**C**) Calculation of Id3 expression in ctrl and BMP4 treated C17.2 mouse stem cells. *** *p* < 0.001. (**D**) Calculation of BrdU positive cells in ctrl and BMP4 treated C17.2 mouse stem cells. * *p* < 0.05. (**E**) Representative images of Id3 and BMP4 colocalization in the SVZ and SGZ of 14 MO female mice. Scale bar, 50 μm. (**F**) Representative images of Id3 expression in the SVZ and SGZ of female mice at 2, 8, 12 and 18 MO. Scale bar, 50 μm. (**G**) Western blotting analyses of proteins extracted from the SVZ and SGZ of three mice at 2, 8, 14 and 28 MO. β-actin is used as a loading control. (**H**,**I**) Relative quantification of Western blotting analysis of Id3 levels in the SVZ (**H**) and SGZ (**I**) of female mice at 2, 8, 14 and 28 MO. Data is represented as the mean protein intensity normalized to β-actin ± S.E.M. from 3 independent mice. * *p* < 0.05, ** *p* < 0.01 and *** *p* < 0.001. (**J**) Representative images of Id3 expression in the SVZ and SGZ of female mice at 10 MO after saline, BMP4 and noggin infusion. Scale bar, 50 μm. (**K**) Western blotting analyses of proteins extracted from the SVZ and SGZ of three mice at 10 MO after saline, BMP4 and noggin infusion. β-actin is used as a loading control. (**L**,**M**) Relative quantification of Western blotting analysis of Id3 levels in the SVZ (**L**) and SGZ (**M**) after the various treatments. Data is represented as the mean protein intensity normalized to β-actin ± S.E.M. from 3 independent mice in each group. ** *p* < 0.01. (**N**) Representative images of BrdU and DCX positive cells in the SVZ and SGZ from 9 MO female mice ten days after mice were grafted twice with lentivirus expressing negative control (NC) and wild type Id3 (Id3-WT). Scale bar, 50 μm. (**O**) Quantification of BrdU and DCX positive cells in the SVZ from 9 MO female mice ten days after mice were grafted with lentivirus expressing negative control (NC) and wild type Id3 (Id3-WT). * *p* < 0.05 and ** *p* < 0.01. (**P**) Quantification of BrdU and DCX positive cells in the SGZ from 9 MO female mice ten days after mice were grafted with lentivirus expressing negative control (NC) and wild type Id3 (Id3-WT). * *p* < 0.05 and ** *p* < 0.01.

To figure out how BMP4 exerts its inhibitory effect on adult neurogenesis, we first checked BMP4 downstream genes. Mouse neural stem cell line C17.2 was exposed to 100 ng/mL of BMP4 for 12 h, after which the mRNA levels of Id1-4 were determined. BMP4 treatment significantly increased Id3 mRNA, decreased Id4 but did not change the Id1 and Id2 transcription (Figure 3A and Supplemental Figure S2A). Meanwhile, BMP4 induction for 12 h resulted in ~29.4% decrease in BrdU incorporation (Figure 3B,D) and markedly upregulated Id3 expression (Figure 3B,C), suggesting that Id3 may play a role in the suppressed NSC proliferation caused by BMP4 treatment.

We then investigated the expression pattern and the dynamic changes of Id3 in both SVZ and SGZ. In the SVZ, intense staining of Id3 was identified in the lateral side of the ventricle, while in the SGZ, Id3 staining was mostly identified in the SGZ and hilus (Figure 3E). Id3 was expressed mainly in Nestin^+^GFAP^+^ NSCs and NeuroD1^+^ neural progenitor cells (Supplemental Figure S2B). Immunofluorescence staining of mice brains also demonstrated the colocalization of BMP4 and Id3 in NSCs in both neurogenic regions (Figure 3E). Furthermore, we found that Id3 increased gradually with age in both SVZ and SGZ (Figure 3F,G), which was in line with the BMP4 expression pattern. Western blotting results showed that compared to 2 MO, the protein level of Id3 in 28 MO mice was increased three to four-fold in the SVZ while the increase was much higher in the SGZ than in the SVZ (Figure 3H,I). Therefore, the spatial and temporal expression profiles of Id3 further support Id3 as a relevant target of BMP4 and suggest its involvement in age-related neurogenesis.

To check whether Id3 acts as a downstream target of BMP4 to modulate adult neurogenesis in vivo, we adopted BMP4 and noggin infusion in 10 MO female mice. Both immunostaining (Figure 3J) and Western blotting (Figure 3K–M) results demonstrated that BMP4 infusion led to a drastic Id3 enhancement compared to the control group, while noggin infusion brought about a marked Id3 reduction in both neurogenic regions, suggesting that BMP4 modulated neurogenesis via Id3 induction.

To further explore the association of Id3 with adult neurogenesis, we stereotaxically injected lentiviruses expressing negative control (NC) or wild type (WT) Id3 into the SVZ and SGZ of 9 MO female mice. Id3 was found to be elevated in both SVZ and SGZ ten days after lentivirus injections (Supplemental Figure S2C), confirming its ectopic expression. The overexpression of Id3 significantly inhibited adult SVZ and SGZ neurogenesis (Figure 3N). Compared to the control group, the number of BrdU^+^ and DCX^+^ cells in the SVZ decreased ~38% and ~33%, respectively (Figure 3O), whereas in the SGZ it decreased ~34% and ~32%, respectively (Figure 3P). Taken together, these results indicated that Id3 might be a potential target of canonical BMP signaling to exert inhibitory effects on adult neurogenesis.

### 3.4. Id3 Inhibited NeuroD1 to Restrain Progenitor Cell Differentiation

**Figure 4:**
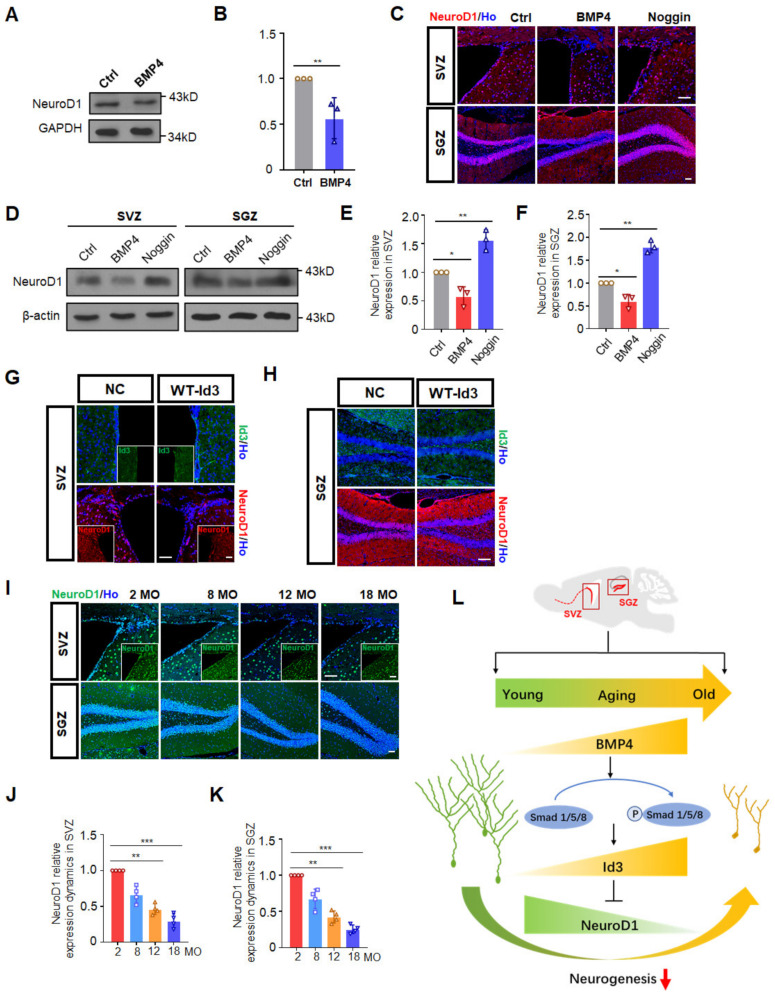
Id3 repressed NeuroD1 to restrain neurogenesis during aging. (**A**,**B**) Western blotting analysis (**A**) and protein quantification (**B**) of NeuroD1 in ctrl and BMP4 treated C17.2 mouse stem cells. GAPDH is used as a loading control. Data is represented as the mean protein intensity normalized to GAPDH ± S.E.M. (*n* = 3). ** *p* < 0.01. (**C**) Representative images of NeuroD1 expression in the SVZ and SGZ of female mice at 10 MO after saline, BMP4 and noggin infusion. Scale bar, 50 μm. (**D**) Western blotting analyses of proteins extracted from the SVZ and SGZ from three mice at 10 MO after saline, BMP4 and noggin infusion. β-actin is used as a loading control. (**E**,**F**) Relative quantification of Western blotting analysis of NeuroD1 levels in the SVZ (**E**) and SGZ (**F**) after the various treatments. Data is represented as the mean protein intensity normalized to β-actin ± S.E.M. from 3 independent mice in each group. * *p* < 0.05 and ** *p* < 0.01. (**G**) Representative images of Id3 and NeuroD1 immunostaining in the SVZ of 9 MO female mice ten days after mice were grafted with lentivirus expressing negative control (NC) and wild type Id3 (Id3-WT). Scale bar, 50 μm. (**H**) Representative images of Id3 and NeuroD1 immunostaining in the SGZ of 9 MO female mice ten days after mice were grafted with lentivirus expressing negative control (NC) and wild type Id3 (Id3-WT). Scale bar, 100 μm. (**I**) Representative images of NeuroD1 expression in the SVZ and SGZ from female mice at 2, 8, 12 and 18 MO. Scale bar, 50 μm. (**J**) NeuroD1 relative expression dynamics in the SVZ of 2 MO, 8 MO, 12 MO and 18 MO female mice. ** *p* < 0.01 and *** *p* < 0.001. (**K**) NeuroD1 relative expression dynamics in the SGZ of 2 MO, 8 MO, 12 MO and 18 MO female mice. ** *p* < 0.01 and *** *p* < 0.001. (**L**) Working model of the study. Age-related increase in BMP signal causes inhibition of neurogenesis in SVZ and SGZ. Canonical BMP signal activation during aging causes Id3 accumulation, which further inhibits NeuroD1, leading to defective differentiation and decreased neurogenesis.

As impaired dendritic arborization of newborn neurons in the SGZ was observed upon BMP4 treatment, we next explored the possible underlying mechanisms responsible for this defective differentiation. Id3, a transcriptional regulator that has a typical helix-loop-helix (HLH) domain, could bind with the basic HLH domain to mitigate expression of some pro-neural transcription factors. Among them, NeuroD1, a bHLH transcription factor, is known as a target gene of Id proteins that is critical for neuronal differentiation. We then examined the effect of BMP4 on NeuroD1 expression after co-culturing with BMP4 in vitro and by BMP4 infusion in vivo. NeuroD1 expression in C17.2 mouse stem cells was markedly decreased by treatment with BMP4 (Figure 4A,B). BMP4 and noggin infusion in 10 MO female mice also showed that BMP4 caused decreased NeuroD1 expression in both SVZ and SGZ, while noggin totally rescued that decline (Figure 4C–F). We also detected NeuroD1 expression after WT-Id3 lentivirus injections and discovered that NeuroD1 declined after Id3 overexpression in the SVZ (Figure 4G) and SGZ (Figure 4H), indicating that NeuroD1 expression was negatively regulated by Id3 in the NSCs. In support of this, NeuroD1 expression declined progressively in NSCs of SVZ and SGZ with age, which was negatively correlated with Id3 expression dynamics (Figure 4I–K). As NeuroD1 is a pro-neural marker, the decreased level of NeuroD1 after BMP4 treatment or by overexpression of Id3 would be expected to lead to a decreased neuronal differentiation.

Taken together, these results indicated that the increased activation of canonical BMP-Smad1/5/8 and its downstream Id3-NeuroD1 axis contributed to declined neurogenesis during aging.

## 4. Discussion

In recent years, several vital signaling pathways have been identified in the regulation of NSC self-renewal, proliferation and differentiation as well as migration and functional integration [14,31]. Crosstalk between these signaling pathways constituted precise and sophisticated regulation of adult neurogenesis [30]. Previous studies reported conflicting results regarding the role of BMP signal in adult SVZ neurogenesis. Whereas some studies report an inhibited neurogenesis, others report a promoted neurogenesis [23,32]. To resolve inconsistencies in the literature regarding BMP4 and neurogenesis, the present study has specifically addressed the interplay between age-related BMP4 levels and adult neurogenesis in both SVZ and SGZ. Notably, our finding that the increase in BMP signaling with age is consistent with the observations of Yousef et al. [21], showing that the age-related increase in BMP signaling in the hippocampus inhibited neurogenesis. However, this study was confined mainly to the SGZ and analyzed only two time points. Moreover, they established that the decreased hippocampal neurogenesis with age was attributed to the BMP-mediated signaling via Smad1 but failed to identify a specific downstream target. In the present study, we extended the time points to four different senescence stages ranging from 2 MO to 28 MO, which revealed a more dynamic change during aging, and found that canonical BMP4 signal increased with age and inhibited neurogenesis in both neurogenic regions. Importantly, we have identified Id3 as a major target of BMP4 in NSCs of SVZ and SGZ, which exhibited a similar increase during aging. The accumulated Id3 in the progenitor cells in aged mice strongly inhibited the neurogenic transcription factor NeuroD1, mitigating determination and differentiation of neurons (Figure 4I). To clarify the correlation between BMP4 levels and adult neurogenesis, we demonstrated that intracerebroventricular infusion of different doses of BMP4 to middle-aged mice activated Smad1/5/8 phosphorylation and upregulated Id3 expression, which further restrained NeuroD1, leading to attenuated neurogenesis in both neurogenic regions and defective differentiation in the SGZ. Therefore, our findings revealed that changes in BMP signaling underlie the decrease in neurogenesis with age and identified Id3 as a potential therapeutic target in the aged nervous system.

The BMP signal not only induced Id4-mediated NSC quiescence [28] but also affected the Id3-mediated differentiation cascade of neural progenitors at an early stage. The four members of the Id family share similar domains but with different N-terminal and C-terminal domains, which determine their different protein interactions [33]. Though the dominant mechanism of Id proteins relies on the direct interaction and modulation of tissue-restricted bHLH factors such as NeuroD1 and Ascl1 in the brain, the mode of action of these four small proteins is highly complex, so whether other Id proteins play a role as a downstream effector of BMP signal remains unclear.

Adult neurogenesis in the mammalian brain is dynamically regulated by a number of signaling pathways in the local NSC microenvironment, including BMPs and WNTs. The BMP and WNT signaling pathways interact mutually at multiple levels via the binding of both SMAD4 and TCF/LEF transcription factors to the promoter and enhancer of some target genes like Emx2 and Msx2 [34,35]. The interaction between these two niche signaling pathways remains very unexplored. Previous studies have identified the role of a transcription factor LEF1 in the mechanistic convergence of the BMP and WNT pathways [36]. NeuroD1 is a key effector of Wnt to promote adult neurogenesis and survival of neuronal progenitors [37,38]. Our data demonstrated that NeuroD1 and Id3 are negatively correlated with each other upon BMP4 and noggin treatment and during the aging process as well, indicating that Id3 acted as not only an effector of BMP4 but also a negative regulator of NeuroD1 in vivo, and NeuroD1 might be a convergence point between the BMP and the WNT routes. However, how Id3 affected NeuroD1 expression remains unclear. Ids have been reported to inhibit gene transcription by sequestration and forming dimers with E proteins like Tcf4 and Tcf3 [39,40], it is possible that E proteins are involved in the Id3-induced inhibition of NeuroD1. Further studies will be needed to test this possibility.

Aging is a vital co-variable regulatory factor affecting neurogenesis and causing notable neurodegenerative disorders and brain tumors [41]. Those neurodegenerative diseases caused by aging are great burdens for the families and systems of care and even the whole of society. Considering Id proteins are involved in important cellular events related to tumorigenesis and cancer progression [42,43], different approaches to reduce aberrant Id protein and restore differentiation of proliferative cells have been successfully developed and applied. For example, some antisense oligonucleotides, siRNAs and microRNAs targeted to decrease Id protein have been validated to reduce tumor growth, invasiveness and metastasis [44,45]. Our finding that the age-associated increase in BMP signaling underlies the decrease in neurogenesis through increased expression of the transcription factor Id3, makes Id3 a promising therapeutic target in the aged nervous system which may lead to a return to normal neurological function.

## 5. Conclusions

In summary, we revealed that the BMP signaling pathway is upregulated with age to mitigate neurogenesis. Id3 accumulation in the neurogenic areas caused by BMP signal activation restrained NeuroD1 in progenitor cells to hinder neuronal differentiation. Our findings not only clarify the correlation between BMP4 levels and adult neurogenesis, but also identified Id3 as a potential and prospective therapeutic target in the aged nervous system.

## Figures and Tables

**Table 1:** Primer list for quantitative RT-PCR.

Name	Forward Primer (5′-3′)	Reverse Primer (5′-3′)
GAPDH	TGCACCACCAACTGCTTAGC	GGCATGGACTGTGGTCATGAG
Id1	TGAACGTCCTGCTCTACGAC	TTGCTCACTTTGCGGTTCTG
Id2	GAAAGCCTTCAGTCCGGTGA	TGGTCCGACAGGCTGTTTTT
Id3	GCCCGAGAGAAGGACTGAAC	CGACACCCCATTCTCGGAAA
Id4	TCCCGCCCAACAAGAAAGTC	CTGTCTCAGCAAAGCAGGGT

## Data Availability

Not applicable.

## Supplementary Materials

The following supporting information can be downloaded at: https://www.mdpi.com/article/10.3390/biomedicines10051147/s1, Figure S1: Noggin promotes neurogenic differentiation in the SGZ; Figure S2: In vitro and in vivo characterization of Id-NeuroD1 expression.
