# Supplementary material for: BMP4 Exerts Anti-Neurogenic Effect via Inducing Id3 during Aging
Source: Biomedicines. 2022 May 17;10(5):1147. doi: 10.3390/biomedicines10051147 (PMC9138880; doi:10.3390/biomedicines10051147)
Supplement: Supplementary file 1 [file biomedicines-10-01147-s001.zip › revised supplementral figures.pdf]

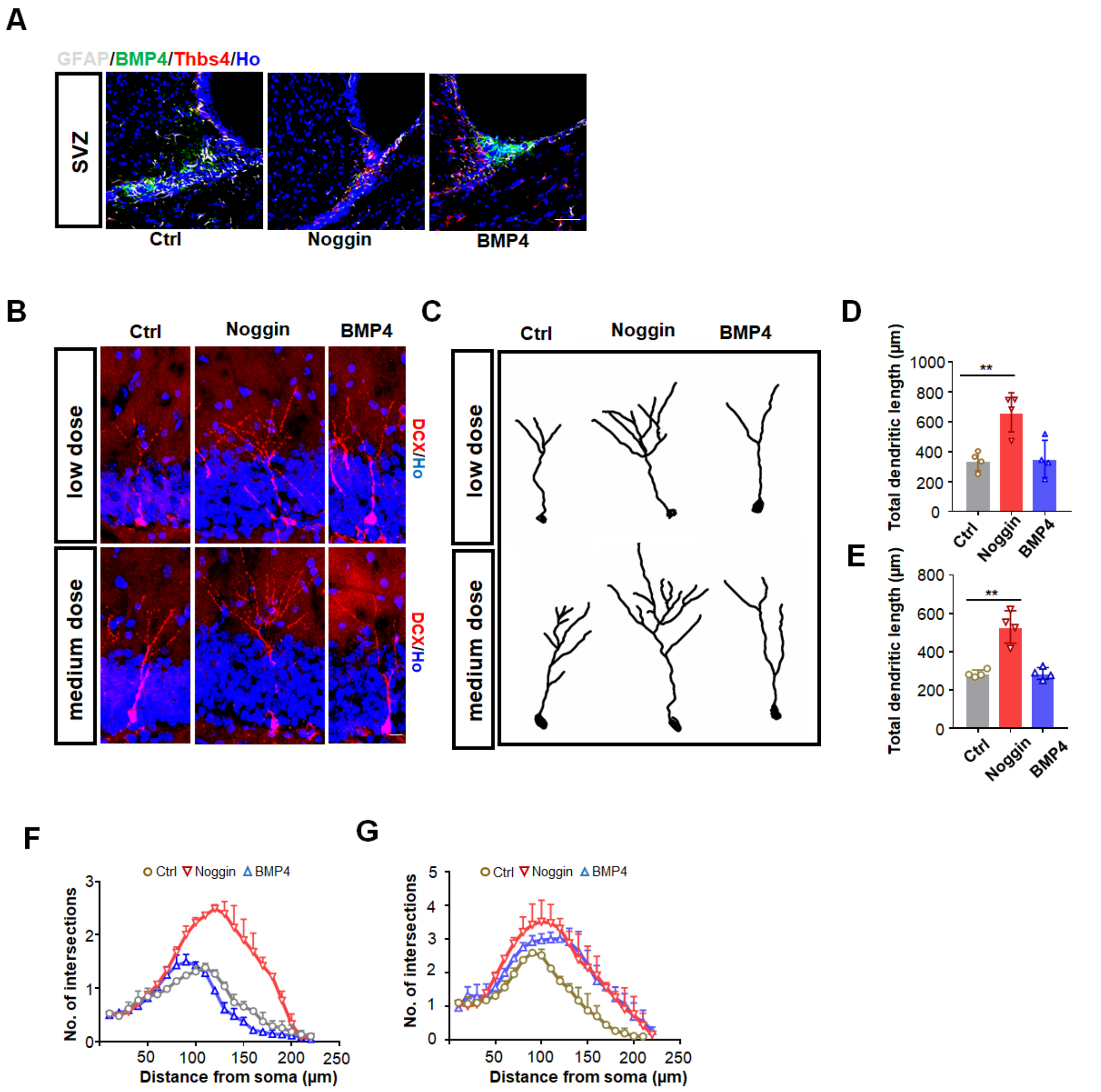

Supplemental Figure S1. Noggin promotes neurogenic differentiation in the SGZ. (**A**) Representative images of GFAP, Thbs4 and BMP4 staining in the SVZ of 8 MO female mice one week after saline, noggin or BMP4 infusion. Scale bar, 50 μm. (**B**) Representative images of the morphology of DCX-positive immature neurons in the SGZ of 8 MO female mice one week after saline, a low (top panel) and a medium (bottom panel) dose of noggin or BMP4 infusion. Scale bar, 10 μm. (**C**) Image tracing of DCX positive immature neurons in the SGZ of 8 MO female mice one week after saline, a low (top panel) and a medium (bottom panel) dose of noggin or BMP4 infusion. Scale bar, 10 μm. (**D, E**) Quantification of the total dendritic length of DCX positive immature neurons in the SGZ of 8 MO mice one week after saline, a low **(D)** and a medium **(E)** dose of noggin or BMP4 infusion. n = 20-25 neurons from 4 mice. $^{**}p < 0.01$ (**F, G**) Quantification of dendritic complexity of DCX-positive immature neurons in the SGZ of 8 MO mice one week after saline, a low (**F**) and a medium **(G)** dose of noggin or BMP4 infusion. n = 20-25 neurons from 4 mice.

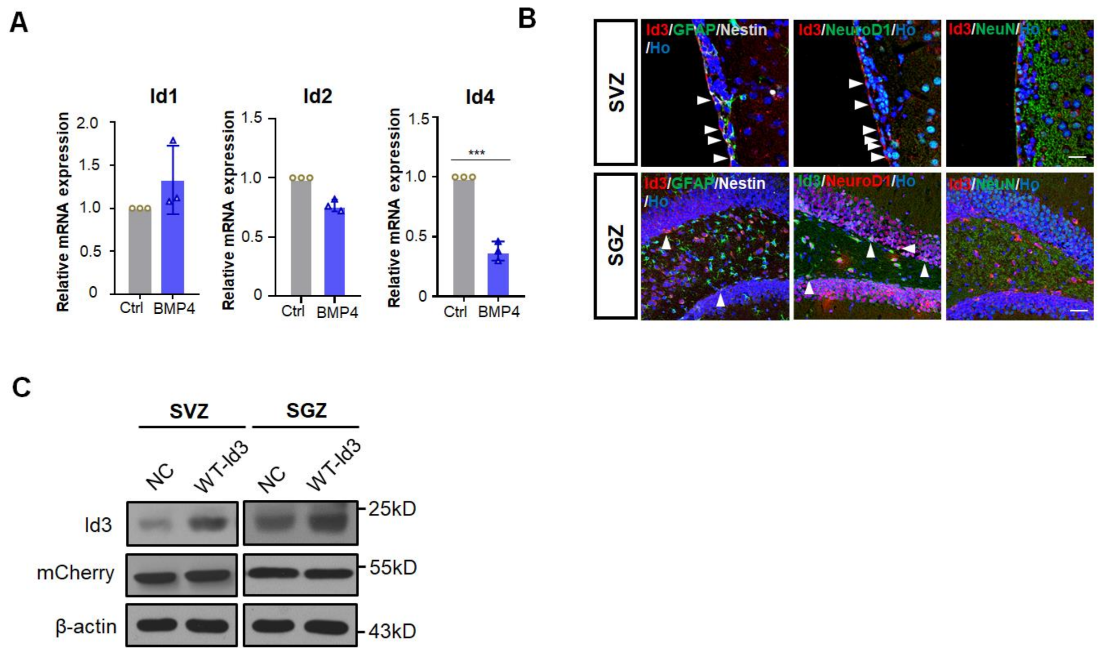

Supplemental Figure S2. In vitro and in vivo characterization of Id-NeuroD1 expression. **(A)** Transcription levels of Id1, Id2 and Id4 in ctrl and BMP4 treated C17.2 mouse stem cells analyzed by qPCR. n = 3. ***$p < 0.001$ **(B)** Characterization of Id3 positive cells in the SVZ (top panel) and SGZ (bottom panel) of 14 MO female mice. White arrowheads in the top panels indicate $Nestin^{+}Id3^{+}GFAP^{+}$ NSCs and $Id3^{+}NeuroD1^{+}$ TAPs of SVZ. White arrowheads in the bottom panels indicate $Nestin^{+}Id3^{+}GFAP^{+}$ NSCs and $Id3^{+}NeuroD1^{+}$ IPCs of SGZ. Scale bar, 50 μm. **(C)** Western blot analyses of proteins extracted from the SVZ and SGZ of three mice grafted with lentivirus expressing negative control (NC) and wild type Id3 (Id3-WT). β-actin is used as a loading control.
